# Addressing Inequities in Urban Health: Do Decision-Makers Have the Data They Need? Report from the Urban Health Data Special Session at International Conference on Urban Health Dhaka 2015

**DOI:** 10.1007/s11524-016-0046-9

**Published:** 2016-05-16

**Authors:** H. Elsey, D. R. Thomson, R. Y. Lin, U. Maharjan, S. Agarwal, J. Newell

**Affiliations:** NCIHD, University of Leeds, Leeds, Yorkshire UK; Department of Social Statistics and Demography, University of Southampton, Southampton, UK; The ARK Foundation, Dhaka, Bangladesh; Health Research and Social Development Forum (HERD), Kathmandu, Nepal; Urban Health Resource Centre (UHRC), New Delhi, India

## Abstract

Rapid and uncontrolled urbanisation across low and middle-income countries is leading to ever expanding numbers of urban poor, defined here as slum dwellers and the homeless. It is estimated that 828 million people are currently living in slum conditions. If governments, donors and NGOs are to respond to these growing inequities they need data that adequately represents the needs of the urban poorest as well as others across the socio-economic spectrum.

We report on the findings of a special session held at the International Conference on Urban Health, Dhaka 2015. We present an overview of the need for data on urban health for planning and allocating resources to address urban inequities. Such data needs to provide information on differences between urban and rural areas nationally, between and within urban communities. We discuss the limitations of data most commonly available to national and municipality level government, donor and NGO staff. In particular we assess, with reference to the WHO’s Urban HEART tool, the challenges in the design of household surveys in understanding urban health inequities.

We then present two novel approaches aimed at improving the information on the health of the urban poorest. The first uses gridded population sampling techniques within the design and implementation of household surveys and the second adapts Urban HEART into a participatory approach which enables slum residents to assess indicators whilst simultaneously planning the response. We argue that if progress is to be made towards inclusive, safe, resilient and sustainable cities, as articulated in Sustainable Development Goal 11, then understanding urban health inequities is a vital pre-requisite to an effective response by governments, donors, NGOs and communities.

## Introduction

In this report, we present an overview of the need for data on urban health for planning and allocating resources to address urban inequities. Such data needs to provide information on differences between urban and rural areas nationally, between and within urban communities. We discuss the limitations of data most commonly available to national and municipality level government, donor and NGO staff. We present two innovative approaches to improve the quality of the data. Finally, we assess how these approaches have the potential to improve responses to urban poverty. This information was originally presented at a special session at the International Conference on Urban Health, Dhaka, 2015.

Rapid and uncontrolled urbanisation is evident across the majority of low and middle-income (LMIC) countries. This growth is particularly evident in South Asia where urban populations are projected to rise from 45 to 62 % by 2050.^1^ Governments are struggling to respond to this scale of growth. The infrastructure—housing, sanitation, health care, education, fuel, electricity, roads—needed to support this expanding population is rarely available, particularly for the poorest. For one third of the world’s urban population, 828 million people, this means living in slum conditions^1^. These conditions are fuelling the deepening trend of inequities across a wide range of health and social outcomes.^2^

National level decision-makers, local governments, donors and communities in low income countries need data to understand and respond to these inequities, and particularly the needs of the poorest in urban and rural areas. We believe that current data frequently overlooks the urban poorest, defined in this paper as the homeless and those living in slum conditions^1^ whether in informal settlements or in rented permanent dwellings, sometimes dispersed among better-off households,^3^ making them invisible to planners and decision-makers.

Currently available data comes from several sources: Census data; routinely collected clinical data and cross-sectional household surveys. All these data sources have limitations. Our assessment highlights how the urban poorest are frequently absent from the data sources available to decision-makers at this macro level. Census data is commonly collected every 10 years; in the context for rapid urbanisation and highly transient urban poor populations, such data soon becomes out of date. Furthermore, censuses exclude the homeless and settlements that are seen as illegal.^3^ This situation is exacerbated by the length of time it can take for official lists of slums to be updated, leaving many slums unrecognized for years.^4^ Recent enumeration work in five Indian cities by the Urban Health Resource Centre (UHRC) found 40% of slums were unlisted and therefore unrecognized, this equates to 36% of all slum residents.^5^

Routinely collected clinical data aids understanding of the scale and trend of diseases and service use. However, clinical data excludes those managed by private medical practitioners, pharmacies, NGOs and traditional providers, thus underestimating prevalence and service use. Data is rarely disaggregated beyond male and female and provides no details on any other patient demographics such as age, level of poverty or home location. Publically available data is frequently aggregated to district or regional level and thus does not support small area planning. The move to electronic medical records has the potential to improve this data source considerably.^6^

Cross-sectional household surveys are a vital addition to the data available to decision-makers. Over the last twenty years or more, approaches and questionnaires have become standardized for many surveys allowing comparison across countries and over time. Over 200 Demographic and Health (DHS) and a similar number of Multiple Indicator Cluster Surveys (MICS) have been conducted since programmes began in 1984 and 1995, respectively.^7^^,^^8^

Whilst cross-sectional surveys have large sample sizes (between 5000 and 30,000 households), they do not collect samples of sufficient size to compare inter-urban or intra-urban disparities. In addition there are four methodological challenges which could lead to under-representation of the urban poorest and skew urban estimates in household surveys. Firstly, census data is commonly used to determine sampling frames, but this excludes ‘illegal’ settlements and the homeless.^3^

Secondly, inconsistent definitions of urban and rural may mean that peri-urban poor are miscategorized as rural; particularly when slums have burgeoned beyond the government defined urban boundaries.^9^ A third challenge is that household listing maps, produced during the second stage of sampling by survey implementers, often assume one dwelling is occupied by one household. Although questionnaires are designed to include non-typical residents including servants and extended family, they overlook whole households that share a dwelling. Multiple household dwellings may include households that split residence, for example with a dwelling in a rural village, households not listed on rental contracts, or households that view their residence as temporary as is common in poor neighborhoods.

A fourth challenge is the definition of a household. Many surveys follow the DHS definition of a household as “a person or group of related and unrelated persons who usually live together in the same dwelling unit(s) or in connected premises, who acknowledge one adult member as the head of the household, and who have common cooking and eating arrangements”.^10^ This definition can become problematic in urban areas: for example, in many of Dhaka’s slums several families share a cooking pot; whilst in Kathmandu, several individuals, often single men, share rooms with no cooking facilities, eating instead at street vendors. This multiple occupancy presents a challenge for survey enumerators who may not be aware of the poorest occupants within a dwelling.

Understanding inequities may also be constrained by the approach to assessing differences in wealth. DHS wealth quintiles are the most commonly used relative measure of wealth (see Tables 1 and 2). The measure is based on household ownership of physical assets such as water source type and cell phone ownership. They are calculated separately for urban and rural populations and then combined to account for the different value of the same asset in a rural versus urban context.^11^ Within urban areas, using physical assets to measure differences in wealth can prove misleading. Wealth includes income, saving, access to credit, and other financial assets beyond physical assets. For the poorest urban dwellers, high rents can keep a household in crippling poverty. Our work in Nepal highlights how those in some of Kathmandu’s informal settlements pay little or no rent and may be comparatively better-off than those living in better constructed formal dwellings paying high rents. These nuances are overlooked by a purely assets based categorisation.

**TABLE 1:** Publically available reports and datasets Nepal: urban HEART indicators covered and measures of wealth

	Survey (latest available)	Representation	Core (12) urban HEART indicators covered	Other urban HEART indicators covered	Measure of wealth
Nepal	Demographic and Health Survey (2011)	National Sub-national Urban–rural Women Men Children	• Infant mortality rate • Access to safe water • Access to improved sanitation • Skilled birth attendance • Fully immunized children • Prevalence of tobacco smoking • Unemployment • Completion of primary education	• Under 5 mortality rate • Solid fuel use • Literacy • Underweight children	Asset-based wealth quintiles
	Multiple Indicator Cluster Survey (2010)	National Sub-national Urban–rural Women Children	• Access to safe water • Access to improved sanitation • Skilled birth attendance • Completion primary education	• Underweight children • Breastfeeding • Solid fuel use • Literacy • Teenage pregnancy	Asset-based wealth quintiles
	Global Youth Tobacco Survey (2011)	National Youth only	• Tobacco prevalence		None
	Nepal Living Standards Measurement Survey (2010–2011)	National Urban/rural	• Access to safe water • Access to improved sanitation • Unemployment • Completion of Primary Education	• Women in the workforce • Poverty • Secure tenure • Morbidity for cancers, CVD, respiratory disease • Solid fuel use • Literacy	Consumption quintiles

**TABLE 2:** Publically available reports and datasets Bangladesh: urban HEART indicators covered and measures of wealth

	Survey (latest available)	Representation	Core (12) urban HEART indicators covered	Other urban HEART indicators covered	Measure of wealth
Bangladesh	Demographic and Health Survey (2011)	National Sub-national Urban–rural Women Men Children	• Infant mortality rate • Diabetes • Access to safe water • Access to improved sanitation • Skilled birth attendance • Fully immunized children • Prevalence of tobacco smoking • Unemployment • Completion of primary education	• Under 5 mortality rate • Solid fuel use • Literacy • Underweight children • Overweight and obesity • Breastfeeding • Teenage pregnancy	Asset-based wealth quintiles
	Urban Health Survey (2013)	Urban	• Infant mortality rate • Access to safe water • Access to improved sanitation • Unemployment • Skilled birth attendance	• Under 5 mortality rate • Security tenure • Teenage pregnancy • Breastfeeding • Underweight children	Asset-based wealth quintiles
	Multiple Indicators Cluster survey (2012–2013)	National Sub-national Urban–rural Women Children	• Access to safe water • Access to improved sanitation • Skilled birth attendance • Completion primary education	• Underweight children • Breastfeeding • Solid fuel use • Literacy • Teenage pregnancy	Asset-based wealth quintiles
	STEPs: NCD risks Survey (2010)	National Adults	• Prevalence of tobacco smoking • Diabetes	• Physical activity • Overweight and obesity	Asset-based wealth quintiles
	GATS: Global Adult Tobacco Survey (2009)	National urban/rural Adults	• Prevalence of tobacco smoking		Asset-based wealth quintiles

To see whether the research community acknowledges these limitations when using cross-sectional data to understand rural–urban and intra-urban inequities we conducted a search of Global Health Ovid and Medline databases from 2000 to date in September 2015. We used the search terms ‘demographic health survey’ and ‘urban’. After the removal of duplicates, we identified 34 studies that had compared risk factors and outcomes between urban and rural populations. Overwhelmingly these papers find greater risks and worse health outcomes among rural populations when compared to urban populations. Only two of these papers^4^^,^^12^ recommend exploring differences by wealth categories within urban populations. If such surveys systematically under-represent the urban poorest, then the categorisation of ‘urban’ becomes a proxy for ‘wealthy urban’ rather than a representative reflection of the health risks and outcomes of the entire urban population. This bias provides an excessively rosy picture of the health of urban dwellers and masks the conditions and needs of the poorest. The value of disaggregation of urban DHS data as conducted in India by UHRC is highlighted by WHO and UN-Habitat^1^. If governments and donors do not have access to data which represents the needs of the urban poorest, it is unlikely that policies and resource allocation will address urban poverty.

**FIG. 1:**
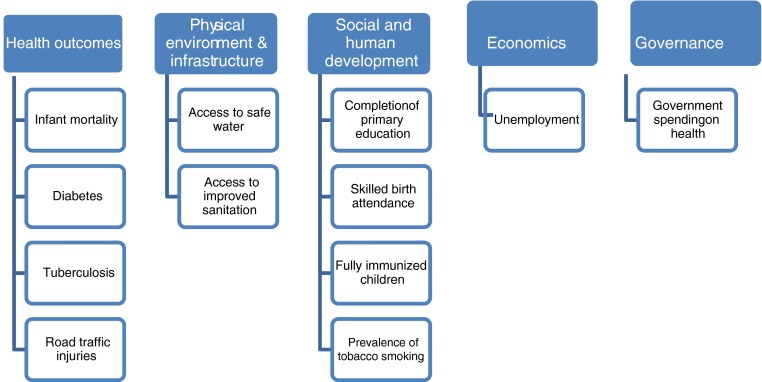
Urban HEART Core Indicators.^13^

To explore the extent to which household surveys can be used to understand urban health and identify health inequities, we assessed publically available reports and datasets of cross-sectional surveys in Bangladesh and Nepal. Six such datasets and survey reports were found (Tables 1 and 2). In order to make this assessment we used WHO’s Urban Health Equity Assessment Response Tool (HEART)^13^ which provides a comprehensive set of 12 core, 18 highly recommended and 6 optional indicators to explore different perspectives of urban health inequities (Fig. 1). The Urban HEART process recommends utilising existing data to assess these indicators. Following the assessment of indicators, the process advocates the involvement of a wide range of government, donor, and civil society stakeholders working together to prioritize and plan the responses to the identified health inequities.

Whilst the available surveys do provide a good coverage of most of the Urban HEART key indicators, particularly DHS, it is also clear that many of the highly recommended and optional indicators are missed. The main purpose of WHO’s Urban HEART process is to provide information to inform a response to inequities within urban areas. Current sampling methods may systematically miss the urban poorest, resulting in biased samples as described above. Further, the sample size of these surveys is insufficient to compare within and between urban inequities. For example, to compare infant mortality,^14^ a HEART indicator that is widely used as a gauge of health system impact on overall child health,^15^ a sample of 1000 to 1500 households of each the urban poorest and urban non-poor would be needed. In the 2011 Nepal^10^ and 2011 Bangladesh DHS,^16^ only 168 and 515 individuals respectively were sampled from the bottom wealth quintile, preventing estimation of HEART indicators in urban areas.

## Innovative Approaches to Improving the Representation of the Urban Poor in Data

### Gridded population sampling in household surveys

To overcome these issues of missing the urban poorest, HERD, and its collaborators including the Ministry of Health and Population in Nepal, adapted several innovations whilst planning an urban health survey. To ensure informal settlements could be included in their survey, the team sampled neighborhoods from gridded population data rather than census data.^17^ Gridded population datasets are based on projected census data disaggregated to small, uniformly-sized areas based on spatial information such as road networks, land cover, and night time lights.^18^ Gridded datasets give ‘un-mapped’ populations a probability of selection and offer population estimates at much smaller geographic units than census data allowing analysis of intra-urban disparities. The R program that we used, GridSamp, is available for free online (https://github.com/ForrestStevens).

To capture a representative sample, including the poorest who share living spaces in what appear to be better-off neighborhoods, HERD strengthened the household listing protocol. In a typical household survey, survey staff hand-map all dwellings in each sampled neighbourhood. HERD increased the speed and accuracy of this process by mapping dwellings directly in OpenStreetMap,^19^ an open source global map, using Android phones with the application OSMAnd [osmand.net]. Approximately half of the dwellings in the survey were already in OpenStreetMap and thousands of additional dwelling locations without any identifying information were integrated into the map. OpenStreetMap is widely used for planning, disaster response, research,^20^ and by community members themselves for advocacy and decision making.^21^ Whilst mapping in the field, the HERD team asked someone in each dwelling, or a neighbour, how many households lived in that dwelling. A household was defined as “a group sharing a cook pot”. These interactions offered the mapping team opportunities to explain the mapping activities to community members. This proved valuable in minimising suspicion and building interest in the follow-on interviewing activities. Whilst talking to community members added time to the household mapping process, use of OpenStreetMap saved time compared to paper-based methods, and we estimate both approaches were comparable in terms of time and cost.

The combination of gridded population sampling and OpenStreetMap household listing were used in a pilot study of 1310 households conducted by HERD in the Kathmandu valley in 2015. We used GridSamp to randomly generate 90 primary sampling areas (PSUs), each with an expected minimum of 146 households. Then mapped households by talking to PSU residents and using OSMAnd in 72 PSUs before 25 April, 2015 when a series of large earthquakes destroyed displaced millions of people in Nepal. This study was cancelled though a future similar study is planned using the same methods. All gridded population datasets have error in the number of modelled population per grid cell. Using WorldPop 100-m grid cells (version 2C)^22^, we found a median of 30 fewer households per PSU than expected during field enumeration (interquartile range: 75 fewer–15 more). Due to variable number of households per PSU, we planned to randomly sample 10% of households from each PSU. Enumerating households in each dwelling proved to be worthwhile as we found a median of 2 households per dwelling (range 1–4). More detailed assessments of the accuracy and feasibility of these methods are needed. This one experience suggests that the budget using GridSamp and OSMAnd was on par with a standard survey using census data and hand-drawn mapping enumeration and it is not any more time consuming in urban areas.

### Combining Assessment and Response using a Participatory Approach

Improving the representation of the urban poor in cross-sectional surveys is vital for improving macro level responses. However, such data does not help with micro level local planning within different districts or wards of a city. The challenges of small area estimations are well known and have been shown to lead to inaccurate estimates of disparities across populations and areas.^23^ The extent of this lack of local area data are highlighted by the experience of Urban HEART which has been used successfully in several high and middle-income countries, but in low income settings the full urban HEART process, including the ‘R’ for response has only happened within pilot sites.^24^ The lack of availability of local area data either from cross-sectional surveys or routine clinical data to compare different neighborhoods or wards is one factor that has undermined the implementation of HEART in low income settings. In response UHRC, India worked in partnership with networks of slum-level community groups and local stakeholders to develop, at low cost, a participatory version of urban HEART.

Building on earlier work,^4^^,^^5^ and at the request of WHO SEARO^24^ the UHRC team simplified the HEART indicators and assessment process. The approach was first used in Indore and Bally and further refined in Agra during 2014–2015. A key aspect of the approach involved facilitating slum community groups to identify the response or action in parallel with the assessment.

The process followed by UHRC involved four main steps. Firstly, slum community group members and UHRC’s social facilitators developed simple actionable indicators based on Urban HEART’s four categories of housing and physical Infrastructure; social, health and human development; economics and governance. Secondly, teams of local women’s group members were trained on the indicators and approach using simple non-technical language in spoken Hindi. The women then assessed the indicators across different neighborhoods through consultation with other women’s group representatives. The assessment was conducted using a three-colour scale of red, yellow or green marks to depict status of deprivation; this can be easily understood regardless of literacy levels.

Consultation meetings were then held across clusters of five to seven slums to develop the response plan based on the ideas that had emerged during the assessment process.

The perseverance and confidence of the women, based on their careful analysis of the problems within their communities, enabled them to advocate for changes. The use of community petitions, reminders to Municipal Corporation, and gentle persuasion enabled improvements to governance and accountability. In Agra from 2014 to 2015, several concrete improvements in slum areas resulted from this advocacy, for example 6300 people benefitted from improved community water supply; 40,000 from paved streets; 34,000 from cleaning of drains and 60,000 from the installation of a sewage system. Further, the sustained efforts of these confident community groups enabled 20,000 people to gain government proof of address and a picture ID card. These are of critical importance as they are a requirement for claiming entitlements, services and social benefits which would otherwise be unattainable by members of these communities. The women’s groups were particularly keen to improve maternity services and with polite requests and coordination with health department officials they were able to increase women’s access to institutional deliveries with 3942 women delivering in government and affordable private hospitals and 1101 accessing the government’s Maternity Benefit Scheme (Janani Suraksha Yojana). Immunisation rates also improved with 1,460 children being immunized at government health services. The women also triggered local actions including building bridge over large open drains, reductions in the number of drinking and gambling joints and increased access to savings and credit groups managed by trained slum women’s group representatives. These schemes have supported over 500 families to keep their children in school, and over 400 families store grain at harvest time, a measure to address food insecurity during low (or no) wage-earning times.

This approach brings the knowledge and wisdom of slum women’s groups to the fore, allowing them to prioritize the actions needed for an immediate and effective responses. Some examples of the actions prioritized by the women include: community sensitization rallies against alcoholism and gambling; removal of alcohol and gambling joints; petitions to local government for cleaning of garbage and drains; petitions requesting installation of water supply in several slums and the provision of picture IDs and address-proof for 500 people allowing their access to free education. The study shows a practical approach to build/strengthen self-reliance and resilience among vulnerable segments of city populations. It also demonstrates how regular mentoring and training can build collective confidence and the skills (such as negotiation skills) required to address vulnerabilities and access to services and entitlements.

## Conclusions

As WHO’s Urban HEART^13^ emphasises, using existing data to inform the response to urban inequities is preferable to conducting more expensive surveys. However, current nationally representative household surveys face several limitations in identifying and assessing the health needs of the urban poor. Furthermore, there is very limited data to inform small area responses at ward level. The work presented during our special session at ICUH 2015 highlighted not only the problems of existing data sources but also the great potential of new approaches such as gridded sampling techniques to improve the representation of the urban poorest within household surveys. The use of crowd-sourcing applications, such as OpenStreetMap, offers great potential to plug the gaps in traditional approaches to mapping populations for household survey samples. This can provide a verifiable dataset which local authorities, NGO and communities can easily update and utilize for action.

It is easy to become focused on the ‘assessment’ component of Urban HEART and overlook the ‘response’. The participatory adaptation of Urban HEART from UHRC provides a practical working methodology to assess health inequities across neighborhoods and wards to provide information for governments and NGOs engaged in slum health programming. Importantly the participatory approach gives equal weight to the Response with the identification of immediate actions to be taken by residents and by local governments and other providers.
